# [Expression of Concern] PI3K/Akt signaling pathway-induced heme oxygenase-1 upregulation mediates the adaptive cytoprotection of hydrogen peroxide preconditioning against oxidative injury in PC12 cells

**DOI:** 10.3892/ijmm.2025.5609

**Published:** 2025-08-18

**Authors:** Liqiu Mo, Chuntao Yang, Mofa Gu, Dongdan Zheng, Lin Lin, Xiuyu Wang, Aiping Lan, Fen Hu, Jianqiang Feng

Int J Mol Med 30: 314-320, 2012; DOI: 10.3892/ijmm.2012.1002

Following the publication of this paper, it was drawn to the Editor's attention by a concerned reader that, for the cell viability experiments shown in Fig. 2E, the 'Control' image (top row) and the 'ZnPP' image (bottom row) appeared to show an overlapping section of data, such that data which were intended to have shown the results of differently performed experiments appeared to have been derived from the same original source. The authors were contacted by the Editorial Office to offer an explanation for the apparent anomaly in the presentation of the data in this paper, although up to this time, no response from them has been forthcoming. Owing to the fact that the Editorial Office has been made aware of potential issues surrounding the scientific integrity of this paper, we are issuing an Expression of Concern to notify readers of this potential problem while the Editorial Office continues to investigate this matter further.

